# *Wickerhamomyces* Yeast Killer Toxins’ Medical Applications

**DOI:** 10.3390/toxins13090655

**Published:** 2021-09-15

**Authors:** Laura Giovati, Tecla Ciociola, Tiziano De Simone, Stefania Conti, Walter Magliani

**Affiliations:** Department of Medicine and Surgery, University of Parma, 43126 Parma, Italy; laura.giovati@unipr.it (L.G.); tecla.ciociola@unipr.it (T.C.); tiziano.desimone@unipr.it (T.D.S.); walter.magliani@unipr.it (W.M.)

**Keywords:** killer yeasts, killer toxins, antimicrobial activity, medical applications, *Wickerhamomyces anomalus*

## Abstract

Possible implications and applications of the yeast killer phenomenon in the fight against infectious diseases are reviewed, with particular reference to some wide-spectrum killer toxins (KTs) produced by *Wickerhamomyces anomalus* and other related species. A perspective on the applications of these KTs in the medical field is provided considering (1) a direct use of killer strains, in particular in the symbiotic control of arthropod-borne diseases; (2) a direct use of KTs as experimental therapeutic agents; (3) the production, through the idiotypic network, of immunological derivatives of KTs and their use as potential anti-infective therapeutics. Studies on immunological derivatives of KTs in the context of vaccine development are also described.

## 1. Introduction

Competition for space, nutrients and other resources in the environment is a rather common mechanism through which different species of microorganisms can interact with or prevail over others, determining the composition of microbial communities within different ecological niches. The production and excretion of molecules with toxic activity, such as bacteriocins and yeast killer toxins, can be included among the mechanisms by which some microorganisms can harm or kill their competitors, contributing to natural selection.

Bacteriocins are ribosomally synthesized antimicrobial peptides produced from a wide variety of bacteria that inhibit the growth of similar or closely related bacterial strains. Broad-spectrum bacteriocins have also been described. Based on their structure, mode of action, mechanism of biosynthesis and self-immunity, bacteriocins could deserve serious consideration as potential alternatives to traditional antimicrobials for use in agriculture, food storage, veterinary and even human medicine [1,2,3].

A similar phenomenon of competition is present in yeasts, based on the production of killer toxins (KTs, or mycocins) that are secreted proteins or glycoproteins capable of killing susceptible microorganisms with various mechanisms of action, through interaction with specific superficial receptors. Interestingly, killer yeasts are immune to their own KTs. Since the first description of the “killer phenomenon” in *Saccharomyces cerevisiae*, by Bevan and Makower in 1963 [4], more than 100 different species of yeasts belonging to more than forty genera, including basidiomycetes and ascomycetes, have been described as killer yeasts, thus attesting to the widespread diffusion of the phenomenon and its ecological relevance [5,6]. Numerous studies over the years have greatly contributed to clarifying the molecular characteristics of various KTs, their physiology and mode of action and the genetic determinants encoding for their production. As relatively simple eukaryotic cells, killer yeasts have also represented a valid model to study relevant aspects of eukaryotic cell biology, such as mechanisms of processing and extracellular secretion of proteins. Comprehensive reviews have been published on these subjects to which the reader is referred [5,6,7,8,9,10,11,12].

Although the spectrum of antimicrobial activity of KTs was initially considered limited to susceptible cells belonging to the same species as the producing yeast or to closely related species, it is now known that some KTs may be active against a great variety of eukaryotic and prokaryotic microorganisms. Taxonomically unrelated fungi, bacteria, protozoa and even achlorophyllous saprophytic algae are among the described KT-susceptible strains, including spoilage and pathogenic microorganisms involved in relevant plant, animal and human infections [6]. The mode of action, which still needs to be clarified for many KTs, is variable and includes cell wall or membrane damages as well as arrest of the cell cycle [10].

As natural antimicrobials, KTs and the producing killer yeasts have been exploited for possible applications in food production and preservation as well as in biological control of plant, animal and human pathogens [5,10,13]. Some KTs produced by *Wickerhamomyces anomalus* and other species belonging to the genera *Pichia* and *Williopsis* have aroused particular interest for potential applications in the medical field.

## 2. *Wickerhamomyces anomalus* Killer Strains and *Wa*KTs

In the last two decades, there has been a continuous reclassification of yeasts, including KT-producing strains, into new genera and species. For example, based on nuclear DNA reassociation studies and phylogenetic analysis of gene sequences, species of the genus *Hansenula* have been reassigned to the genus *Pichia* and then to the genus *Wickerhamomyces* [14,15,16,17]. Among the species belonging to this genus, *W. anomalus* has aroused particular interest in microbiology and biotechnology fields, food production and biopreservation, as well as the development of innovative therapeutics, due to its specific characteristics, adaptation properties, frequent detection in natural environments (plants, soil, fruits, animals) and involvement in various fermentation processes [18]. Another key feature of *W. anomalus* is its capability to produce and secrete KTs, characterized by a broad spectrum of activity, comprising relevant plant, animal and human pathogens [19,20]. Many KTs exert their optimal activity at acidic pH and temperatures below 30 °C, which could be a problem for biomedical application [5]. Over the years, various KTs produced by *W. anomalus* (named *Wa*KTs or, previously, *Pa*KTs and *Ha*KTs) have been described [21,22,23,24]. Notably, some killer strains can produce more than one toxin with different characteristics [25]. The most attractive features of some of these chromosomally encoded KTs [26] are the broad spectrum of activity and their mechanism of action, mediated by the interaction with specific cell wall receptors. In most cases, 1,3 or 1,6 β-glucans are the potential receptors and/or targets of KTs, sometimes characterized by exo-β-glucanase activity [21,24,27,28,29].

## 3. Killer Toxins’ Medical Applications

Three possible medical applications of *W. anomalus* and related species killer strains or their KTs have been tested and suggested: 1. direct use of killer yeasts as biological competitors; 2. direct use of KTs as potential antimicrobial molecules with broad activity; 3. production and use of immunological derivatives of KTs. In the following paragraphs, killer strains and KTs for these applications are described (summarized in Table 1).

### 3.1. Direct Use of Killer Yeasts

The direct application of some killer yeasts as biocontrol agents against mold and bacteria in the agro-food field has been suggested by many authors [58,59]. In the medical field, a more recent interesting proposal concerns the use of toxin-producing *W. anomalus* strains in the symbiotic control of arthropod-borne diseases [60]. In particular, a *W. anomalus* killer strain (*Wa*F17.12) has been isolated from the murine malaria vector *Anopheles stephensi*. *Wa*F17.12 has been reported to secrete a glycoproteic KT of about 140 kDa, characterized by a strong anti-plasmodial activity against *Plasmodium berghei* that causes malaria in rodents [30,31]. This KT inhibited early plasmodial sporogonic stages (ookinetes), causing several morphological and structural alterations, thus severely compromising the parasite survival. Its antimicrobial action has been associated with a β-glucanase activity, responsible for the hydrolysis of β-glucans located in the parasite cell membrane. Additional studies confirmed the ability of KT to damage the parasite also in vivo, thus preventing its development in the mosquito midgut and its possible transmission by the biological vector [32,33]. Since symbiotic strains of *W. anomalus* have been isolated in several vector species [61,62], further studies will hopefully clarify whether the produced KTs are also capable of interfering with other arthropod-borne pathogens. These observations suggest the potential benefits of this killer yeast-based approach in preventing the transmission of relevant vector-borne diseases.

### 3.2. Direct Use of Killer Toxins

Although most KTs have a very narrow spectrum of activity, some produced by *W. anomalus* and *Williopsis saturnus* var. *mrakii* (previously known as *Hansenula mrakii*) have a broader spectrum. 

Many years ago, Polonelli et al. first isolated a killer strain (UP25F, now ATCC 96603) of *Pichia anomala* (now *W. anomalus*) able to produce a KT (*Wa*96603KT) characterized by a broad spectrum of activity, including Gram-positive and Gram-negative bacteria, in addition to yeasts and filamentous fungi [34]. Although not yet fully understood, the mechanism of action of *Wa*96603KT seems to involve β-1,3 D-glucans as the potential receptors and/or targets. A possible direct use of *Wa*96603KT as a therapeutic agent has been investigated in different models of experimental infection, such as pneumocystosis in SCID mice, by in vitro attachment tests and in vivo infectivity assays, [35] and superficial *Malassezia furfur* infection in guinea pigs [63]. Despite the positive results of such experimental approaches, the use of *Wa*96603KT as a systemic therapeutic agent was prevented by the physico-chemical features of the molecule. Therapy by parenteral administration was unfeasible because of *Wa*96603KT ineffectiveness at the physiological pH and temperature conditions. Moreover, due to the high molecular weight (220 kDa), *Wa*96603KT proved to be strongly antigenic. Furthermore, when perfused in a rat small intestine model, *Wa*96603KT induced secretion and severe acute injury due to ischemic degeneration of villi and sloughing of surface epithelium [64].

Another interesting, wide-spectrum KT (HM-1) has been identified in *W. saturnus* var. *mrakii* IFO 0895. This KT, composed of 88 amino acids with five disulfide bridges, proved to inhibit yeast cells in the growing stage without being toxic to mammalian cells [41,44]. HM-1 is believed to bind to a receptor in the yeast cell wall, then to a putative receptor on the cell membrane and finally inhibit β-1,3-glucan synthase with a cytocidal effect in sensitive cells [42,43]. Even if HM-1 proved to be highly stable in a wide range of temperature and pH values (between 2 and 11), its possible direct clinical use was excluded for its antigenicity to the host.

Panomycocin is another wide-spectrum KT, active also against human dermatophytes and *Candida* spp., which is produced by the *W. anomalus* killer strain NCYC 434. It is a 49 kDa monomeric glycoprotein with an exo-β-1,3-glucanase activity, and it is able to kill target cells by disrupting their cell wall. It has exactly the same amino acid sequence as the exo-β-1,3-glucanase produced by *W. anomalus* strain K [21,28,49,50]. In an attempt to minimize the in vivo adverse effects of the molecule and to obtain therapeutic preparations to be envisaged at least for topical use, panomycocin has been encapsulated in a biocompatible, biodegradable and nontoxic lipid mixture similar to the lipid composition of the epidermal stratum corneum. In this formulation, panomycocin displayed a strong in vitro anti-*Candida* activity. When tested on a commercially available reconstructed human vaginal epithelium model, experimentally infected with *Candida albicans* and *C. glabrata* vaginal isolates, the formulation was strongly fungicidal [51]. Further studies were conducted to enhance panomycocin thermal stability at body temperature. By site-directed mutagenesis, a changed toxin has been designed to be stable for potential topical therapeutic purposes in an acidic environment, such as the vagina [52].

Other KTs produced by *W. anomalus* strains isolated from plants, marine environment, milk, or insect microbiota showed to exert direct antimicrobial activity on different yeast species, including azole-resistant clinical isolates of *C. albicans* and *C. glabrata* [24,53,56,57], malaria parasites [32] and pathogenic bacteria, such as *Listeria monocytogenes* and multidrug-resistant strains of *Acinetobacter baumannii* [54,55]. 

Overall, the results obtained in the studies on the direct use of KTs as antimicrobial therapeutics leave many questions open, with particular regard to their in vivo activity, stability, toxicity and safety. As outlined before, the reported KTs are usually high molecular weight proteins/glycoproteins, therefore highly antigenic. A possible solution to this issue could be provided by new technological approaches. Analysis of KT sequence and changes in amino acid composition could allow the identification of the molecule’s active site to find inside the sequence of KT small peptides still retaining the antimicrobial activity. Selvakumar et al. [65], on the basis of the amino acid sequence, synthesized thirteen overlapping peptides from the primary structure of HM-1. Scanning analysis of these peptides by immunoblotting, surface plasmon resonance and yeast growth inhibition assays identified a peptide, named P6 (^39^TGGSTDGKQG^48^), which could be part of the active site of HM-1, as it was able to strongly inhibit the growth of the tested fungi (*S. cerevisiae*, *Candida* and *Cryptococcus* species). Further studies indicated the presence of a binding peptide sequence inside P6 (^41^GSTDGK^46^), identifying D-44 and K-46 as essential amino acids for the killing activity. To clarify the extent to which these observations may have an impact on a possible therapeutic use of these peptides, further investigations must necessarily be envisaged.

A significant feature of all the previously described KTs is their interaction with/inhibition of β-1,3-glucans, which are lacking in mammalian cells, thus representing specific targets for potential antimicrobial therapy, as discussed below.

In an attempt to overcome many of the issues related to unfavorable physico-chemical features and toxicity, our group and others have exploited the idiotypic network to obtain immunological derivatives (antibodies and peptides) of some KTs capable of mimicking their antimicrobial activity.

### 3.3. Production and Use of Immunological Derivatives of Killer Toxins

**Figure 1:**
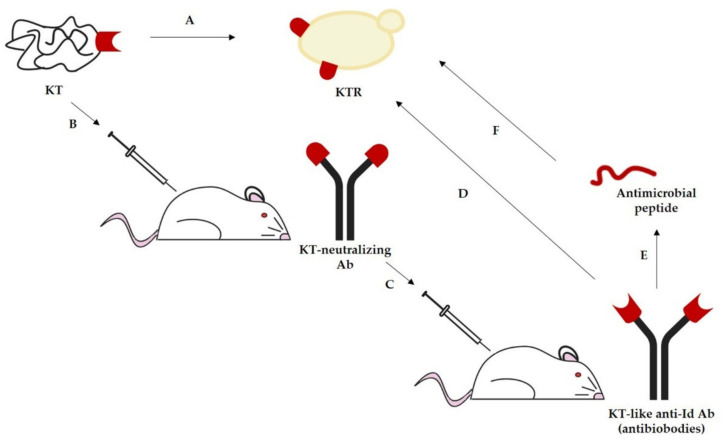
Application of the idiotypic network theory to the yeast killer phenomenon to obtain active immunological derivatives of KTs. (A) KT exerts a direct antimicrobial activity following interaction with the KT receptor (KTR) present on susceptible microorganisms. (B) Immunization with KT elicits neutralizing antibodies directed against the KT functional epitope. (C) The idiotype of a KT-neutralizing antibody, mimicking the KTR, may act as a vaccine eliciting anti-idiotypic antibodies. (D) Anti-idiotypic antibodies, whose idiotype mimics the KT functional epitope (antibiobodies), act as antimicrobial molecules through interaction with KTR on susceptible microorganisms. (E) Peptides derived from the binding site of antibiobodies may maintain the antimicrobial activity against KT-susceptible microorganisms (F).

The idiotypic network theory, in the case of KTs, would predict that the interaction between the functional epitope of a toxin and its specific receptor (KTR) in sensitive microbial cells should be mirrored by the binding between the idiotype (Id) of a KT-neutralizing antibody (Ab) and its anti-Id. This postulates that Id may mimic the KTR, thus acting as a vaccine, and anti-Id may mimic the active site of KT, thus acting as an antimicrobial molecule (Figure 1).

In this context, for many years, our research group has been studying the previously mentioned *Wa*96603KT produced by *W. anomalus* ATCC 96603. As already highlighted, *Wa*96603KT was characterized by a very broad spectrum of antimicrobial activity, but it proved to be unusable as a potential therapeutic agent, except for topical use, due to its toxicity, ineffectiveness at physiological conditions and antigenicity. The goal was to obtain, through the idiotypic network, immunological derivatives of *Wa*96603KT still capable of exerting the antimicrobial activity of the toxin. Several studies have been carried out over the years, whose results are reviewed in [37,39]. This research has also made it possible to explore immunological aspects beyond the field of KTs, suggesting new perspectives on the anti-infective potential of Abs and their peptide derivatives, as briefly described below [66,67].

It all started with the initial production of a monoclonal Ab (mAb KT4), capable of neutralizing *Wa*96603KT. Then, mAb KT4 was used as an immunogen in animals to elicit polyclonal anti-Id Abs. These anti-Id Abs, purified by affinity chromatography, were able to recognize and bind to specific KTRs on the surface of microbial cells. In yeasts, KTRs are preferentially located on germ tubes and budding scars, where β-glucans are exposed. Even more interesting was the observation that these Abs, defined as “antibiobodies” (Killer Abs, KAbs), could mimic the antimicrobial activity of *Wa*96603KT, thus acting as antibiotics [37]. These initial observations suggested the potential of two different experimental anti-infective approaches: Id vaccination and anti-Id therapy. 

Id vaccination is based on the use of mAb KT4, or similar KT-neutralizing Abs representing the internal image of KTRs, as immunogens to elicit KAbs in vivo. Anti-Id therapy is based on the direct use of KAbs or their derivatives as therapeutics. In fact, parenteral and mucosal Id vaccination with mAb KT4 proved to protect animals against experimental fungal infections [37]. KAbs were also elicited following natural and experimental infections with KTR-bearing microbial cells [68], in particular following immunization with *C. albicans* cells treated with dithiothreitol and protease which expose on the surface β-1,3 D-glucans (i.e., *Wa*96603KT receptors) [69]. A β-1,3 D-glucan-based vaccine, consisting of laminarin, a soluble β-1,3 D-glucan, conjugated to a diphtheria toxoid, elicited the production of protective KAbs and was able to confer active and passive protection against different β-1,3 D-glucan (KTR)-bearing fungi [70,71]. Such a vaccine represents, in theory, the first example of a polyvalent, “universal” vaccine capable of protecting against infections caused by microorganisms bearing β-1,3 D-glucans on their surface. 

On the other hand, the anti-Id therapy approach has been tested for a long time, leading to increasingly intriguing results. First, the production and purification of KAbs of animal or human origin made available macromolecules much more stable than KTs and therefore usable for in-depth studies of their spectrum and mechanism of action. The specificity of KAbs was demonstrated through the neutralization of their activity by mAb KT4. The most relevant issue concerning these KAbs was their qualitative and quantitative availability, depending on their purification from animal or human sources. To overcome this problem, KAbs were produced in monoclonal (mKAb) and recombinant (scFvKAb) formats, characterized by an absolute reproducibility. Thus, a significant activity of these KAbs was demonstrated in vitro and/or in vivo against relevant fungal, bacterial and protozoal pathogens [37,39]. Furthermore, the availability of the sequence of a scFvKAb allowed the production of recombinant KAbs in genetically modified human commensal bacteria, suggesting the possibility of their production in vivo following colonization [72]. 

The analysis of scFvKAb sequence allowed the obtaining of small peptides still able to maintain the biological activity of the whole Ab, thus functionally mimicking *Wa*96603KT. In particular, synthetic peptides reproducing the six complementarity determining regions (CDRs) of scFvKAb and decapeptides obtained from the sequence of ScFvKAb hypervariable region were tested in vitro against *C. albicans*, selected as a model microorganism. The highest candidacidal activity was observed with a decapeptide (P6, EKVTMTCSAS) comprising 3 amino acids of CDR1 L. Alanine scanning of P6 allowed the obtaining of a derivative peptide (killer peptide, KP, AKVTMTCSAS), characterized by an implemented anti-*Candida* activity [36]. The ease of obtaining KP in unlimited quantity by chemical synthesis allowed the testing of its activity in vitro and/or in vivo against numerous pathogens, both prokaryotic and eukaryotic, in different environmental conditions. KP proved to be active, with different molecular mechanisms, against diverse human pathogens, including yeasts and filamentous fungi (such as *Candida*, *Cryptococcus* and *Malassezia* spp., *Paracoccidioides brasiliensis* and *Aspergillus* spp.), bacteria (Gram-positive and -negative species), protozoa (such as *Leishmania* and *Acanthamoeba* spp., and *Toxoplasma gondii*) and viruses (HIV, Influenza A and Herpes simplex) [38,39,40]. KP also exhibited antimicrobial activity against a broad spectrum of phytopathogenic bacteria and fungi, such as *Erwinia carotovora*, *Pseudomonas syringae*, *Botrytis cinerea* and *Fusarium oxysporum*, and was expressed in planta (*Nicotiana benthamiana*) by using a Potato virus X (PVX)-derived vector. KP-expressing plants were much more resistant to experimental infections by phytopathogens and could be envisaged as very attractive tools for the large-scale production of peptides of interest through molecular farming [39]. In the case of microorganisms, the antimicrobial activity of KP appears to be mediated by its binding to specific receptors, consisting of β-glucans or similar polysaccharide structures, which are absent in mammalian cells. KP is further characterized by an immunomodulatory activity on cells of innate and acquired immunity that may implement its anti-infective potential [38,73]. The antimicrobial, antiviral and immunomodulatory activities of KP also rely on its ability to spontaneously and reversibly self-assemble, in a process catalyzed by β-1,3-glucans or β-glucan-like molecules, as attested by studies on structure-function relationship [38]. Moreover, the study of KP derivatives, obtained by replacing or deleting the first amino acid, demonstrated the possibility of implementing the antimicrobial activity of KP by modification of the amino acid sequence [74]. 

The idiotypic network approach was pursued also by Selvakumar and collaborators, through the production of recombinant anti-Id Abs representing the internal image of the previously described HM-1 KT. ScFvHM-1 Abs were produced by recombinant DNA technology from the splenic lymphocytes of mice immunized by Id vaccination with a mAb neutralizing (nmAb) HM-1. Recombinant Abs were able to exert a broad-spectrum in vitro activity against the tested fungal strains, belonging to *Saccharomyces*, *Candida* and *Cryptococcus* genera, thus mimicking the antimicrobial activity of HM-1. The antifungal activity of scFvHM-1 Abs was neutralized by the HM-1 nmAb, was more potent in inhibiting the yeast growth when compared with HM-1 and was mediated, as for HM-1, by the inhibition of fungal β-1,3-glucan synthase [45,46,47]. Subsequently, Kabir et al. [48], based on the sequence of scFvHM-1 Ab A1, synthesized the peptides representing the six CDRs and other CDR-related peptides and investigated them by Dot blot, surface plasmon resonance analysis and growth inhibition assays. The antifungal activity of the CDR peptides was compared with the one of peptides derived from HM-1 KT. The most active HM-1-derived peptide (P6) inhibited the growth of *S. cerevisiae*, but it was less effective against *C. albicans* and *C. neoformans*. The most active scFv-derived peptide (SP6) was effective against *Candida* and *Cryptococcus* strains through interaction with cell wall β-glucans, as demonstrated by the neutralization of anti-fungal activity with the soluble β-1,3-glucan laminarin. SP6 is a decapeptide, consisting of the first three amino acids of CDR L1 (SVS) and seven amino acids of the framework region 1, as KP. Notably, SP6 (AKVTITCSVS) and KP (AKVTMTCSAS) share 8 of the 10 amino acid residues, both present a wide spectrum of antifungal activity and probably have an identical mechanism of action. 

A logical consequence of the studies on antimicrobial peptides derived from the sequence of recombinant Abs, representing the functional internal image of some KTs, was the assessment of the potential anti-infective activity of other Ab-derived peptides, synthesized as independent molecules and regardless of the specificity of the originating Ab. Indeed, the previously described Ab fragments were characterized by a conserved sequence present in bovine, human, mouse and rabbit recombinant Abs deposited in data banks, with different specificities. Following these observations, numerous studies led our group to achieve interesting results on Ab-derived peptides, quickly moving away from the field of the killer phenomenon, as summarized in dedicated reviews [75,76].

## 4. Conclusions

A direct use of killer yeasts or their KTs as competitors against microbial pathogens is limited by several problems, except perhaps in the interesting option of their exploitation in vector insects for the symbiotic control of arthropod-borne diseases. KT-derived peptides or immunological derivatives of KTs, such as KT-mimicking Abs and their fragments, have been extensively described. KT- and Ab-derived peptides, once selected and tested, can be easily managed in terms of synthesis, quantitative production and sequence modification, in order to improve their activity and delivery systems, in view of a possible therapeutic use. 

In the 21st century, infectious diseases still represent an important challenge for human health, despite the improvements in hygiene, healthcare and socioeconomic status and the extraordinary success of preventative and therapeutic approaches. Globalization and climate changes are favoring the emergence and re-emergence of new and old etiologic agents, often characterized by intrinsic or acquired resistance to anti-infective agents. The growing crowd of immunocompromised or otherwise debilitated individuals represent a further, dramatic challenge for the treatment of infectious diseases. All this severely limits the available therapeutic armamentarium, strongly highlighting the need to develop new therapeutic tools and approaches. In this scenario, KT- and Ab-derived peptides can provide leading structures for the rational design of novel, target-directed compounds applicable, by their own or in synergy with existing agents, in the field of human, animal and even plant infectious diseases.

## Figures and Tables

**Table 1:** Killer strains and KTs for applications in the medical field.

Killer Toxin (KT)	KT Producer	MM (kDa) ^1^	Target	Function	Application	References
*Wa*F17.12	*Wickerhamomyces anomalus* F17.12	140	*Plasmodium berghei*	β-glucanase activity	Killer yeast	[30,31,32,33]
*Wa*96603KT	*W. anomalus* ATCC 96603	220	Bacteria, fungi, protozoa, viruses	Various/nd	KT and derivatives	[34,35,36,37,38,39,40]
HM-1	*W. saturnus* var. *mrakii* IFO 0895	10.7	Yeasts	Inhibition of β-1,3-glucan synthase	KT and derivatives	[41,42,43,44,45,46,47,48]
Panomycocin	*W. anomalus* NCYC 434	49	Human dermatophytes, *Candida* spp.	β-glucanase activity	KT	[21,28,49,50,51,52]
*Wa*1F1-KT	*W. anomalus* 1F1	160–170	*Candida* spp.	β-glucanase activity	KT	[24]
WA40M1, WA45M2 and WA92M3	*W. anomalus* WA40, WA45 and WA92	nd	*C. albicans*, *Acinetobacter baumannii*	nd	KT	[53,54]
KT	*W. anomalus* LMA-827	nd	*Listeria* sp.	Pore formation	KT	[55]
KT	*W. anomalus* YF07b	67	*Candida* spp.	Membrane permeabilization	KT	[56]
Mycocin	*W. anomalus* tp2-15	45, 50	*Candida mesorugosa*	β-glucanase activity	KT	[57]

^1^ MM, molecular mass (kDa); nd, not determined.
